# From molecules to macroevolution: Venom as a model system for evolutionary biology across levels of life

**DOI:** 10.1016/j.toxcx.2020.100034

**Published:** 2020-04-18

**Authors:** Kevin Arbuckle

**Affiliations:** Department of Biosciences, College of Science, Swansea University, Swansea, SA2 8PP, UK

**Keywords:** Organismal vs molecular perspectives, Clinical implications, Venom biology, Venomics, Evolutionary ecology

## Abstract

Biological systems are inherently hierarchical. Consequently, any field which aims to understand an aspect of biology holistically requires investigations at each level of the hierarchy of life, and venom research is no exception. This article aims to illustrate the structure of the field in light of a ‘levels of life’ perspective. In doing so, I highlight how traditional fields and approaches fit into this structure as focussing on describing levels or investigating links between levels, and emphasise where implicit assumptions are made due to lack of direct information. Taking a ‘levels of life’ perspective to venom research enables us to understand the complementarity of different research programmes and identify avenues for future research. Moreover, it provides a broader view that, in itself, shows how new questions can be addressed. For instance, understanding how adaptations develop and function from molecular to organismal scales, and what the consequences are of those adaptations at scales from molecular to macroevolutionary, is a general question relevant to a great deal of biology. As a trait which is molecular in nature and has clearer and more direct links between genotype and phenotype than many other traits, venom provides a relatively simple system to address such questions. Furthermore, because venom is also diverse at each level of life, the complexity within the hierarchical structure provides variation that enables powerful analytical approaches to answering questions. As a result, venom provides an excellent model system for understanding big questions in evolutionary biology.

## Highlights

• Venom is a molecular trait used directly in fitness-relevant ecological interaction.
• Venom is consequently an ideal model system for evolutionary biology.
• A ‘levels of life’ perspective is well suited to research in venom biology.
• This structure of the field provides many advantages to guide future studies.
• Clinical implications can arise from studies of venom at all levels of life.

## 1 Venom at the interface of molecular biology and ecology

Venoms have long captured the interests of evolutionary biologists; in the early 1900s Alcock and Rogers (1902) were already investigating how snake venoms could have originated under Darwinian natural selection. This is perhaps not surprising since venom has convergently evolved many times across the tree of life, has arisen and been modified by evolutionary processes, and has influenced future evolution of venomous groups and their traits (Arbuckle, 2017, Schendel et al., 2019). Nevertheless, the attraction of venom from an evolutionary perspective goes beyond the simple observation that it seems have been a particularly successful trait for a wide range of organisms.

Fundamentally, the adaptive evolution of a (genetically encoded) trait operates on the fitness consequences of variation in phenotypes which result from the underlying genetic variation (Darwin and Wallace, 1858, Futuyma and Kirkpatrick, 2017). The difficulty with many traits is that the link between genetic variation and the fitness consequences of phenotypes can be extremely complex to an extent that reduces the tractability of the system under investigation. For instance, fitness consequences are often highly variable for a given phenotype based on ecological context (sometimes even reversing in sign), a single phenotype is often the outcome of a large number of underlying genes which interact in complex ways, and historical contingency can channel the underlying genetic evolution down restricted routes leading to stochastic evolution of different fitness peaks (Elena and Lenski, 2003). While it is unlikely any natural system can be simple enough to eliminate those limitations, venom does have some advantages.

Venom is a trait for which the phenotype (the resulting toxicological effect) mostly results from very specific interactions between the products of genes (proteinaceous toxin composition) and a target organism (Sunagar et al., 2016). Although this situation has its own complexities at every level, the link between genotype and phenotype is *relatively* straightforward compared to many other traits, giving venom a tractability for studies of evolutionary patterns and processes. Moreover, the complexity at each level (e.g. venom composition or interactions with target animals) combined with the high rate of convergent evolution of venoms at different levels (Arbuckle, 2017) provide a rich source of variation that provides the raw material for hypothesis testing.

In addition to its advantages as a research model *per se*, venom is also well placed as a trait with wide biological relevance at multiple scales. It is quintessentially embedded in the ecological context of the venomous animal because by definition venom is used in interactions with other organisms, whether predators, prey, or competitors (Jackson et al., 2019). Moreover, each of the types of ecological interactions that venom is involved in are closely linked to evolutionary fitness (e.g. energy intake, survival, and competition for mating opportunities), generating a tight linkage between venom phenotypes and fitness. They are also universally characterised as antagonistic coevolutionary relationships (Arbuckle, 2017), which are known or predicted to have widespread and profound impacts on evolutionary dynamics (Queller and Strassmann, 2018). Such effects include increased evolutionary rates of the traits involved as a result of ‘arms races’ and increased lineage diversification rates via escape-and-radiate processes or enhanced ecological opportunity (Ehrlich and Raven, 1964, Schluter, 2000, Arbuckle and Speed, 2015, Broeckhoven et al., 2016). As such, although fundamentally a molecular trait, venom is intricately tied to the ecology and evolution of the organisms in question, providing a powerful example for studies of evolutionary processes and adaptation across biological hierarchies.

In other words, the combination of tractability, variation, and inherent position at the intersection of molecular biology, ecology, and evolution leaves venom as an ideal model system for evolutionary biology. In particular, venom is a prime candidate for evolutionary questions relating to the nature of adaptive evolution of organismal traits across the hierarchy of life. Indeed, this realisation has recently seen a substantial presence in the literature as several recent reviews emphasising the need for and promise of behavioural, ecological, and evolutionary questions in venom research (Calvete, 2013, Valcu and Kempenaers, 2015, Sunagar et al., 2016, Arbuckle, 2017, Arbuckle, 2018, Jackson et al., 2019, Schendel et al., 2019).

## 2 Levels of life and the structure of venom research

The explicit consideration of ‘levels of life’ as an important perspective for thinking about studies of evolution is particularly prominent in the literature on convergent evolution (Losos, 2011, Speed and Arbuckle, 2017). Although the concept is broadly applicable to understanding the structure and consequences of adaptations and other evolutionary changes, the frequency of many-to-one mapping of lower to higher levels explains the focus on convergence (Losos, 2011, Thompson et al., 2017). For instance, different morphological strategies exist to create the functionally convergent dewlap in lizards (Hagman and Ord, 2016), and functional similarity despite different molecular underpinnings seems to be a common scenario (Natarajan et al., 2016, Härer et al., 2018). Such situations are likely to be very common in venom biology as well; many different toxins are possible causes of particular toxicological phenotypes (such as neurotoxicity) and different types of toxicity may be perfectly adequate to perform the same function (e.g. subjugate prey). Hence, taking a levels of life perspective to gain a broad overview of venom research holds great promise for mapping out the field, recognising inherent assumptions, and guiding future research.

**Fig. 1:**
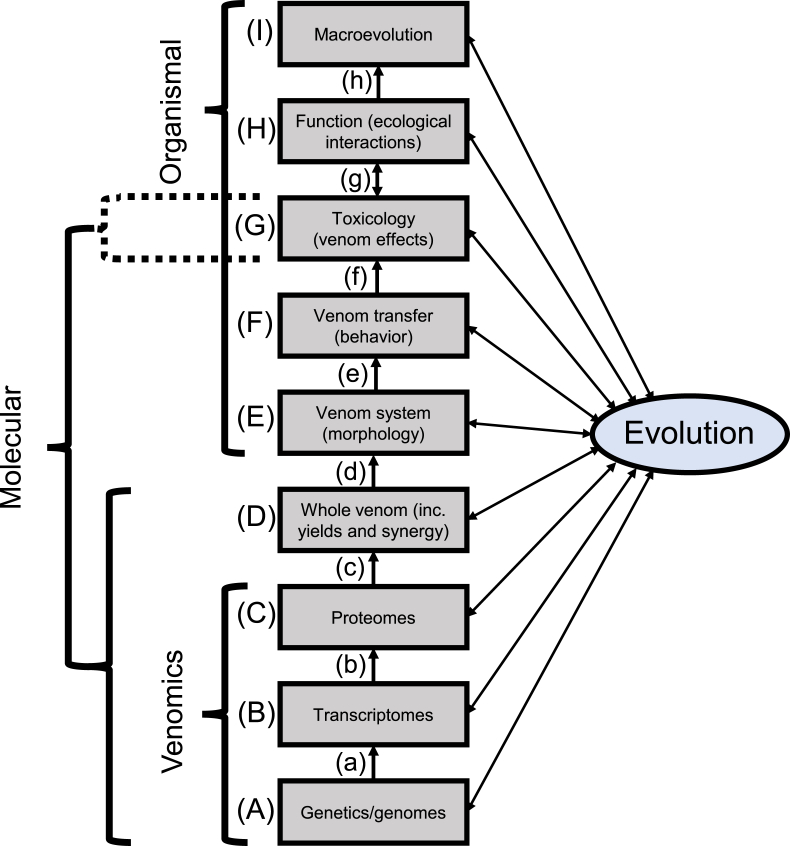
Structure of venom biology as a field, using a levels of life perspective. Levels are denoted by upper case letters, links between levels by lower case letters. Some common descriptors of research (molecular vs organismal biology, venomics) are shown on the left of the figure to illustrate how these map on. Evolution is shown on the right of the figure with links to all levels to highlight that the evolution of traits at every level is possible, and potential evolutionary links exist between any pair of levels; it is a pervasive part of all biology.

An illustrative structure for the field of venom biology is shown in Fig. 1. The basis of this structure is the different levels (denoted by upper case letters) and the links between them (denoted by lower case letters). Importantly, evolution is presented as linking to every level and separately from the central levels to emphasise that, rather than being a level in its own right, it is an integral concept underlying all levels as well as providing potential links between any two levels. For instance, evolution of the proteome (C) can be studied in its own right but there may also be evolutionary links between the proteome and the ecological interactions that give venom its function (H), such as selection around prey-specific toxins (e.g. Bernardoni et al., 2014). Importantly, assessing links between non-adjacent levels (such as proteome, C, and venom function, H) is possible, but inherently makes a range of assumptions (often unappreciated) about the intervening levels and links which are ‘skipped’, and these are made clearer by considering the ‘levels of life’ structure. Where appropriate for ease, precision, and brevity, in the subsequent text I will refer to the levels and links in Fig. 1 by their letters. I will use a shorthand for the links via evolution wherein XY is the relationship between level X and level Y (e.g. link DG is the link between the whole venom and its toxicological effects).

There are two unusual attributes in Fig. 1 that should be highlighted here. Firstly, despite the diagram being hierarchical, in which lower levels are ‘passed’ before higher levels are encountered, there is one two-way arrow at link g. This reflects the fact that although the toxicological effects of venoms lead to their ecological functions (it is those effects which bring on the function), the particular ecological interactions can also have a major influence in venom effects. For instance, toxin resistance is a widespread phenomenon in the animal kingdom (Arbuckle et al., 2017) and results in the particular ecological interaction, e.g. which species you envenomate, determining the venom effects to a potentially large degree. Note that this is different from other levels which can have ‘downward facing’ links via evolutionary relationships, because although toxin resistance itself is an evolved trait, in this case the difference in response is an immediate one that varies by current context. In other words, for a given snake biting a potential prey item, the effect of the venom changes dependent on the species it is interacting with, contrasting with (for instance) the case where proteome changes in response to ecological interactions but only as an indirect result of a prolonged evolutionary response. Second, the dual categorisation of toxicology (G) highlights the strong integration of molecular and organismal approaches, which are both used to study toxicological effects of venoms and provide complementary perspectives. In essence, molecular toxicological studies (typically *in vitro*) investigate the molecular mechanisms of individual toxins, whereas organismal approaches (typically *in vivo*) are more akin to ‘ecological toxicology’ and use whole venoms (or more rarely purified toxins) introduced to whole organisms (ideally natural target organisms or as close a proxy as can reasonably be used).

Importantly, both levels and links in Fig. 1 represent (sub-)fields rather than individual questions *per se*. As researchers we participate in a concerted effort to understand the variation in our favoured levels and/or the links between them. Consequently, the structure presented in Fig. 1 reflects a large-scale, interdisciplinary research programme and demonstrates how the diversity of current research foci link together and inform each other to generate the field we call ‘venom biology’.

## 3 Missing links and the future of venom research

A truly holistic understanding of venom biology must describe and examine the variation present within each level, and also test each link in Fig. 1. However, the attention given to each part of this structure is far from equal. In particular, toxicological effects (G) has historically received the greatest emphasis, and more recently the rise of ‘venomics’ has come to dominate much of venom biology (Escoubas et al., 2008, Calvete, 2013, Valcu and Kempenaers, 2015). While this has resulted in excellent progress for describing variation in venom composition (A-C), venomics has recently been overemphasised relative to other levels (Calvete, 2013), particularly levels and links between D and I (excepting G). Indeed, the ‘omics revolution’ is not unique to venom biology, and Stern (2019) has recently commented on the problems associated with a focus on omics approaches to generate lists but (relatively) neglecting the links and processes generating the discovered variation.

Given that venom is so deeply integrated into organismal biology (it is a fundamentally ecological trait after all, see Section 1) it is unfortunate that studies at the organismal levels have not received as intense study as molecular levels. In fact, with the exception of link g, our understanding of the variation within and links between organismal levels still provides fertile fields for future studies. Macroevolutionary studies of venom (I) have been particularly limited (Arbuckle, 2018), though recently several researchers have started to address venom biology at this scale (e.g. Harris and Arbuckle, 2016, Blanchard and Moreau, 2017, Liu et al., 2018, Barua and Mikheyev, 2019). Even at the molecular level, most focus has been on venomics (or its precursors, A-C) at the expense of strong understanding of the whole venom (D). For instance, we now have data on venom composition for many species of snake, and yet the ecologically important aspect of venom yield is often lacking (Dam et al., 2018, Modahl et al., 2020). Studies aiming to understand the substantial variation in yenom yield are even rarer than those that merely report it (but see Healy et al., 2019), yet the total volume of venom and, remaining on the whole venom level (D), the quantities of different toxins in the venom (combined with their *in vivo* interactions) are likely important factors in venom use and evolution. This again is likely to provide ample scope for future research as it represents a level (and the links involving it) which have received very little attention to date. Indeed, further scrutiny of Fig. 1 will hopefully lead researchers to identify other areas where the depth of our knowledge and understanding is missing, and hence provide a roadmap for where new studies are likely to be particularly fruitful.

## 4 Clinical implications emerge from each level of life

The emphasis of snakebite research has understandably focussed on clinical toxicology, treatment, and epidemiology (Calvete, 2010, Williams et al., 2011, Harrison et al., 2011, Gutiérrez et al., 2017, Longbottom et al., 2018). However, clinical implications can be found at each level of life in my proposed structure for the field of ‘venom biology’, albeit with varying degrees of directness. I do not intend a comprehensive review of these here, but simply highlight a few examples to emphasise the applicability of venom biology across levels of life to clinical toxinology.

The most direct and clearest link is of course toxicology (G), which essentially describes the clinical problem that medics are tasked with resolving. Similarly, proteomics (C) describes the etiological agents of snakebite envenoming, the toxins themselves, and hence have direct importance for clinical management. Genomics (A) can enable identification of possible toxins in an unknown venom based on tissue samples of the animal in question, and hence provide some basis for treatment options in case of future envenomations. Transcriptomics (B) and proteomics (C) provide a clearer picture of what toxins are actually present in the venom and also provide resources for development of new (and in some cases synthetic) antivenom treatments (Calvete, 2010, Williams et al., 2011, Harrison et al., 2011). An understanding of whole venoms (D), including venom/toxin yields and synergistic effects of toxins, will inform predictions of the likely severity of envenomations (Mirtschin et al., 2002) and a fuller range of expected symptoms beyond simple lists of toxins present. The venom system (E) is the animal's means of delivering the venom and hence variations in its depth of penetration, nature of mechanical injuries inflicted, and efficiency of venom delivery will influence the expected prognosis of envenomations (Kardong and Lavin-Murcio, 1993). Snakebite is fundamentally an applied problem in antipredator behaviour, and so defensive behaviour (F) of venomous snakes has clear implications for the epidemiology of snakebite. Despite this few venom biologists are familiar with the broader (and extensive) literature on antipredator defences of animals, even those concerned with snakebite epidemiology. Nevertheless, habitat selection, foraging behaviour, striking kinematics, ‘decisions’ over venom use, and specific behaviours such as venom spitting (in the case of some cobras) are all crucial to understanding the distribution, occurrence, and infliction of snakebite envenoming (F) (Hayes et al., 2002). Understanding the ecological role and use of venoms (H) can also provide important insights into venom (clinical) toxicology, as discussed above in the context of the double-headed arrow at link g. A venom whose evolution has been driven by selection to incapacitate mammals is likely to inflict more severe or more complex envenomations than one selected to incapacitate insects (Davies and Arbuckle, 2019; though more work is needed to confirm these results). Similarly, a venom selected for a defensive function may be particularly effective in causing immediate and intense pain than other venoms (Ward-Smith et al., 2020). Clinical implications of macroevolutionary studies of venom (I) are less frequent and certainly less direct. However, if a broader understanding of the diversification and historical biogeography of venomous species in relation to their venoms is gained (Arbuckle, 2018), insights into the diversity and distribution of such species should help inform epidemiology of envenomations. Moreover, understanding macroevolutionary dynamics and patterns of venom traits will also provide better predictions of envenomations inflicted by less commonly implicated species.

My final point about clinical implications of venom biology is one of needed improvement. In my view, as a community, we could do much better in highlighting the links between our work and snakebite envenomation. There are two common problems here. The first is that we often leave the implications and applications too vague to be useful. Statements towards the end of manuscripts along the lines of ‘our research will improve clinical management of snakebites’ or ‘a greater understanding of [the area covered here] will help reduce the global burden of snakebite envenomations’ are remarkably common. If this is indeed the case, we should make sure we don't stop at such generic statements but clearly explain how our research could be used – otherwise it won't be, and we do a great disservice to all communities involved. The second (though often coincident with the first) common problem is trying to artificially create implications of our research which don't reasonably exist. This is often partially driven by a desire to ‘sell’ our research to grant funders, tenure committees, etc. The reality is that not all studies in venom biology will necessarily have clinical or public health implications, certainly not immediate or direct ones, and that's OK. We should avoid making statements where they are tenuous at best, as they simply muddy the water of what is and is not useful, but where there are applications we should ensure that we clearly explain what they are.

## 5 Conclusion

I have attempted to provide a structure of the broad field of venom biology that is underpinned by considerations of hierarchical levels of life. The intention here is to promote a new perspective on our field, and argue that thinking in terms of levels of life has advantages for better mapping the field, seeing new and poorly explored avenues for future research, and making assumptions of biological links clearer. As a fundamentally molecular trait that directly interacts with other organisms in important fitness-related processes (by definition), venom is ideally suited to understanding broader questions concerning adaptations across levels of life. Consequently, venom biology will hopefully benefit from considering this perspective, and big questions in both molecular and (perhaps especially) organismal biology will benefit by considering venoms as ideal model systems for research.

## Ethical statement

There are no ethical concerns with this work as it is an opinion piece with no animal-based research.

## Declaration of competing interest

The authors declare that they have no known competing financial interests or personal relationships that could have appeared to influence the work reported in this paper.
